# KRT9 is required for GBP5 suppression of human respiratory syncytial virus

**DOI:** 10.1128/jvi.02029-24

**Published:** 2025-01-21

**Authors:** Xinglong Qu, Ziqi Zhu, Xinpei Zhou, Xuehan Wu, Xin Liu, Xiaoyu Sun, Jiayue Zhang, Guanyi Du, Runyu Xue, Qianhua Zhang, Wenyan Zhang, Zhaolong Li

**Affiliations:** 1 Respiratory department of the First Hospital of Jilin University, Changchun, Jilin, China; 2 Institute of Virology and AIDS Research, The First Hospital of Jilin University 664674 , Changchun, Jilin, China; 3 Clinical Medical School, Norman Bethune Health Science 63680 , Changchun, Jilin, China; 4 Department of Infectious Diseases, Infectious Diseases and Pathogen Biology Center, Key Laboratory of Organ Regeneration and Transplantation of The Ministry of Education, The First Hospital of Jilin University 117971 , Changchun, Jilin, China; University Medical Center Freiburg, Freiburg, Germany

**Keywords:** RSV, KRT9, GBP5, SH

## Abstract

Respiratory syncytial virus (RSV) infections continue to plague infants, young children, and older individuals worldwide. Since there is no specific treatment for RSV, characterizing the interactions between RSV and host factors remains crucial for the eventual development of robust therapeutic interventions. In our previous study, guanylate binding protein 5 (GBP5) was shown to promote excessive RSV-small hydrophobic (RSV-SH) protein secretion by microvesicles and inhibited viral replication. In this study, using affinity mass spectrometry, keratin (KRT) 9 was identified to be required for GBP5 to trigger RSV-SH transport. Silencing KRT9 expression reduced the antiviral effects of GBP5 and interferon-γ. A direct interaction was detected between KRT9 and GBP5, but not RSV-SH; a GBP5 binding domain was identified on KRT9. Our results suggest that GBP5, as a bridge, interacts with KRT9 and RSV-SH, after which KRT9 triggers RSV-SH transport. The mechanism underlying the interaction between KRT9 and GBP5 explains the inability of the GBP5-C583A and GBP5-△C mutants in inhibiting RSV replication. Conversely, KRT1, KRT5, and KRT6C, which were identified as potential partners of KRT9, did not affect the GBP5 anti-RSV process. Overall, our study provides evidence for KRT9 involvement in host innate immunity for the first time.

**IMPORTANCE:**

RSV causes severe acute lower respiratory tract infections, which have posed serious health and safety risks to children and older adults worldwide. Although some RSV interventions are available, the longer-lasting monoclonals, which are expensive, are required to be injected before RSV infection, and their protection is observed only up to one RSV infection season; vaccines are currently only available to the elderly but are not suitable for application in infants and young children. As specific drug treatments are absent, a systematic and in-depth mechanism for research is essential. In our study, KRT9 was identified to play an important role in the GBP5 anti-RSV process for the first time. This investigation improved the interaction mechanism between GBP5 and RSV, provided new evidence for the synergistic effect between keratin transport and innate immunity, and opened a new research direction with GBP5 and the keratin transport system as the main subjects.

## INTRODUCTION

Respiratory syncytial virus (RSV) infections trigger severe respiratory illnesses in children younger than 5 years and adults over 65 years of age, having already caused serious health problems and economic burdens globally (1–4). Especially, in recent years, one in every 56 healthy term-born infants is reportedly hospitalized for an RSV infection in Europe (5). As there is a lack of effective drugs to treat these illnesses, an improved understanding of the interaction mechanism of RSV and host innate immunity is urgently needed. RSV belongs to the *Pneumoviridae* family (6). RSV has a single-stranded RNA that consists of 10 genes; these genes produce 11 proteins, specifically nonstructural proteins NS1 and NS2, nucleocapsid proteins N, transcription associated proteins M2-1 and M2-2, phosphoprotein P, large polymerase protein L, surface proteins F and G, matrix protein M, and small hydrophobic protein SH (7). In previous research on anti-RSV, many host factors inhibiting RSV replication were identified. Some of these findings are as follows: Fehrholz *et al*. suggested that APOBEC3G impaired RSV transcription, inhibited replication, and enhanced the mutation rate (8); Busse *et al*. showed that IFI44 and IFI44L restricted RSV replication and transcription (9); Everitt *et al*., Zhang *et al*., and Smith *et al*. reported that IFITM interfered with RSV viral entry and replication (10–12); Mazumder *et al.* found that L13a promoted the formation of a VAIT complex and silenced RSV-M protein translation (13); Han *et al*. reported that CXCL4 blocked RSV binding to HSPGs (14); and in our previous study, we showed that guanylate binding protein 5 (GBP5) inhibited RSV replication via triggering the excessive secretion of viral small hydrophobic (SH) protein by microvesicles (15). In the conventional secretion system, the main secretory proteins contain a signal peptide, which can be bound by the signal recognition particle (SRP), and then translocated into the endoplasmic reticulum (ER) through the translocon SEC61 (16–18). After this, the secretory proteins are exported via ER–Golgi trafficking, which is mediated by COPII and COPI vesicles (19, 20). On the other hand, many cytosolic proteins without the signal peptide (leaderless cargoes) are released through unconventional protein secretion (UPS) that bypasses the ER–Golgi trafficking itinerary (21, 22). However, as an interferon-stimulated gene, GBP5 inhibits the action of multiple viruses including human immunodeficiency virus, severe acute respiratory syndrome coronavirus 2, Middle East respiratory syndrome coronavirus, and Influenza A virus, usually inhibiting glycosylation on viral proteins, furin-mediated cleavage, and reducing the viral envelope locating to the plasma membrane; further, it also reduces the assembly and release of infectious virions. To our knowledge, GBP5 participation in any transport processes by vesicles or fibers has not been reported.

Keratins, the largest subgroup of intermediate filament proteins, represent the most abundant proteins in epithelial cells. Human keratins are encoded by 54 evolutionarily conserved genes, which contain 28 type I and 26 type II genes (23, 24). For assembling a coiled coil heterodimer, type I keratin is matched with a particular type II keratin. The dimers further polymerize into tetramers through interacting along their lateral surfaces in an antiparallel orientation (25, 26). Type I keratin 9 (KRT9) reportedly has three potential type II partners, KRT1 (27), KRT5 (28), and KRT6C (29), and it plays an important role in inducing epidermolytic palmoplantar keratoderma (30–33). In addition, KRT9 expression has been reported to be associated with different types of cancers (34–37). In this study, we found that KRT9 was involved in the GBP5 anti-RSV process, and the interaction between KRT9 and GBP5 was essential for GBP5 anti-RSV function. Furthermore, we identified that the 202–302 fragment of KRT9 played an important role in its interaction with GBP5.

## RESULTS

### Identification of GBP5-associated anti-RSV factor

**Fig 1:**
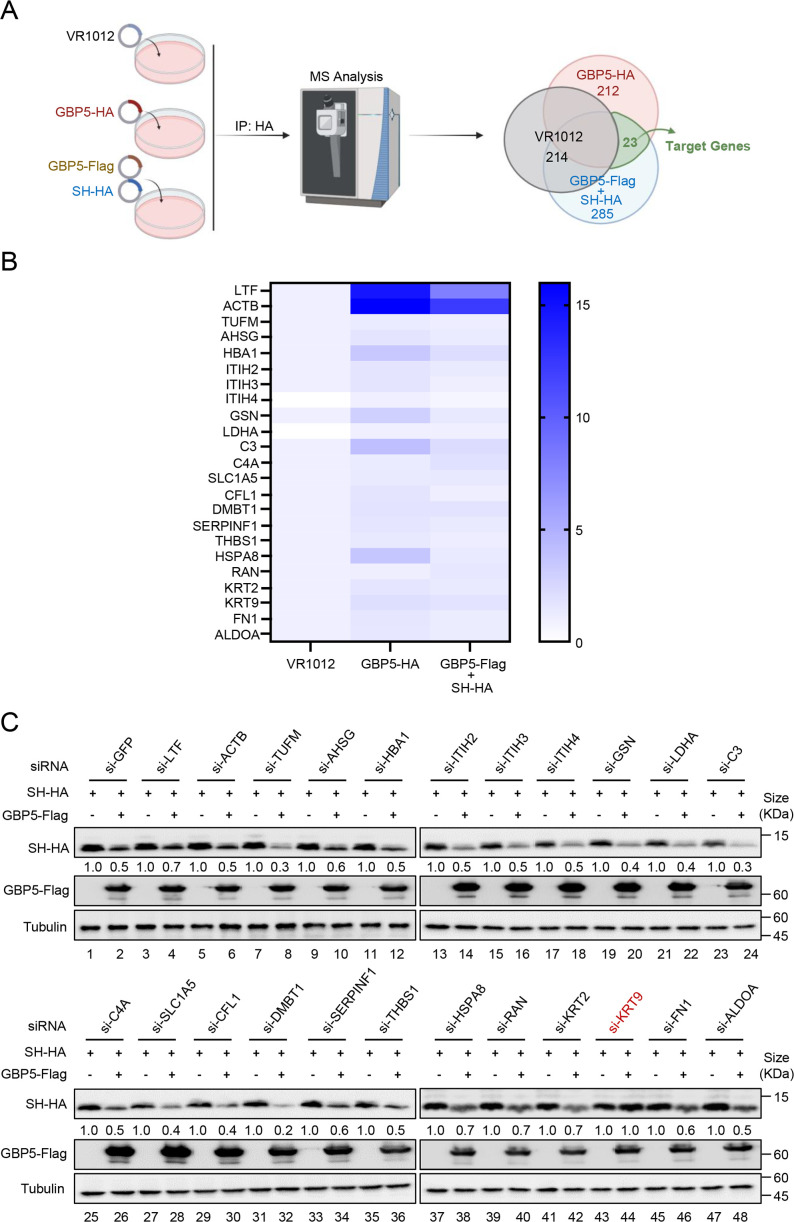
Identification of host factor which is associated with GBP5 inhibiting RSV. (**A**) HEK293T cells transfected with empty vector (VR1012); GBP5-HA or SH-HA combined with GBP5-Flag for 48 hours was harvested and lysed. And then coimmunoprecipitation (Co-IP) was performed with protein-G agarose and anti-HA antibody. The elution from Co-IP was analyzed by MS. Genes appeared more both in GBP5-HA elution (red) and SH-HA combined with GBP5-Flag (blue) thanVR1012 (gray), which might be our target genes (green). (**B**) Binding Strength Analysis (fold-change of VR1012) of the proteins listed in the green area of the venn diagram in (**A**). (**C**) HEK293T transfected with siRNA (synthesized by RiboBio Co. Ltd., Guangzhou, CHN) by Lipofectamine RNAiMAX for 24 hours were transfected with SH-HA combined with the empty vector or GBP5-Flag at the second day for 24 hours. And then, cells were harvested and analyzed by immunoblotting (IB). Tubulin was detected as the internal control. The intensity of SH-HA were analyzed by ImageJ and calibrated by tubulin.

In our previous study, we showed that GBP5 promoted RSV-SH protein release from microvesicles (15). However, GBP5, as a GTPase, was not reported to be associated with the vesicular transport system. These results led us to suspect that other host factors were involved in this transport process. To identify these potential host restrictive factors and reveal the mechanisms of their interactions, we identified the proteomes that interacted with GBP5-HA or SH-HA (with GBP5-Flag present) using co-immunoprecipitation (Co-IP) and mass spectrometry analysis. The common proteins in the two proteomes were selected, and nonspecific binding proteins were excluded by the empty vector (VR1012) proteome (Fig. 1A). After this, 23 potential target genes, which were highly enriched in both the GBP5-HA proteome and the SH-HA combined with GBP5-Flag proteome than the VR1012 proteome, were screened (Fig. 1B). To confirm the host factors, an siRNA library containing the mentioned genes was constructed to detect the associations between GBP5 anti-RSV function. In HEK293T cells, with the library working efficiently (Fig. S1), KRT9 silencing was found to decrease the secretion of RSV-SH triggered by GBP5 (Fig. 1C) and anti-RSV function from GBP5 (Fig. S2). These results suggest that KRT9 could be involved in the GBP5 anti-RSV process.

### KRT9 plays an important role in the GBP5 anti-RSV process

**Fig 2:**
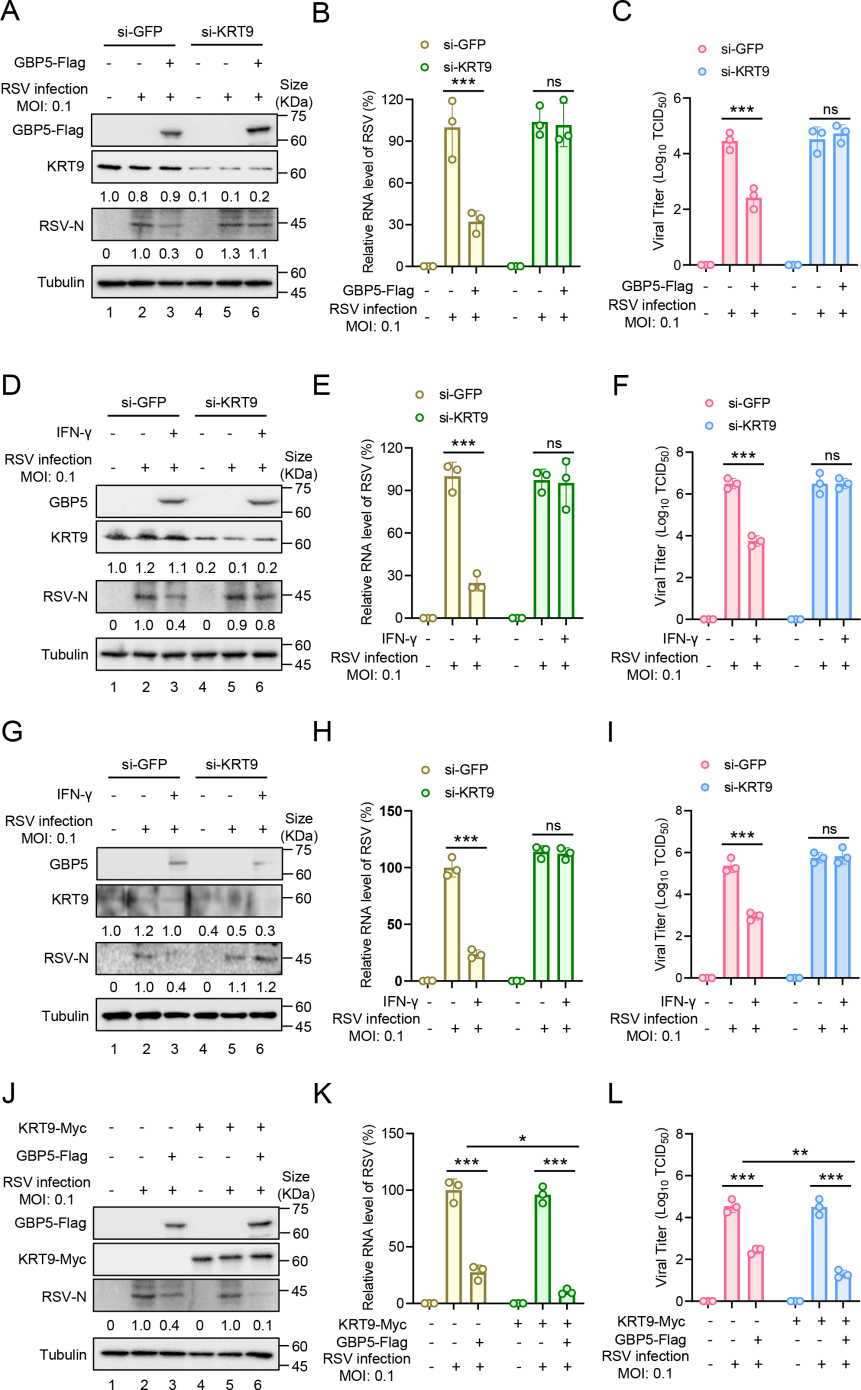
KRT9 is required for GBP5 inhibiting RSV replications. (**A–C**) HEK293T cells transfected with siRNA by Lipofectamine RNAiMAX for 24 hours were transfected with empty vector or GBP5-Flag for 24 hours and then infected with RSV at 0.1 MOI. After 48 hours post-infection, cells were harvested and analyzed by immunoblotting (IB) (**A**) and RT-qPCR (**B**); viral titers in supernatants were measured by the endpoint dilution titer method (**C**). (**D–F**) A549 cells transfected with siRNA by Lipofectamine RNAiMAX for 24 hours were treated with IFN-γ (1,000 U/mL) for 24 hours and then infected with RSV at 0.1 MOI. After 48 hours post-infection, cells were harvested and analyzed by IB (**D**) and RT-qPCR (**E**); viral titers in supernatants were measured by the endpoint dilution titer method (**F**). (G–I) The primary bronchial epithelial cells transfected with siRNA by Lipofectamine RNAiMAX for 24 hours were treated with IFN-γ (1,000 U/ml) for 24 hours and then infected with RSV at 0.1 MOI. After 48 hours post-infection, cells were harvested and analyzed by IB (G) and RT-qPCR (H); viral titres in supernatants were measured by the endpoint dilution titer method (I). (**J–L**) HEK293T cells transfected with KRT9-Myc, GBP5-Flag, and empty vector for 24 hours as annotation were infected with RSV at 0.1 MOI. After 48 hours post-infection, cells were harvested and analyzed by IB (**J**) and RT-qPCR (**K**); viral titers in supernatants were measured by the endpoint dilution titer method (**L**). Data are representative of three independent experiments and shown as average ±SD (*n* = 3). Significance was determined by one-way ANOVA, followed by a Tukey multiple comparisons posttest. *P > 0.05; **P < 0.01; ***P < 0.001; ns means no significance. The intensities of KRT9 and RSV-N were analyzed by ImageJ and calibrated by tubulin.

To confirm the anti-RSV activity of KRT9, siRNA aimed at KRT9 or GFP (used as a negative control) combined with GBP5-Flag or an empty vector were transfected into HEK293T cells. After 24 hours post-transfection, RSV infections were performed at 0.1 multiplicity of infection. Our results indicated that KRT9 knockdown did not affect RSV-N expression without GBP5 expression (Fig. 2A, lanes 2 and 5). However, when GBP5 was expressed in HEK293T cells, KRT9 knockdown eliminated the anti-RSV function of GBP5 (Fig. 2A, lanes 3 and 6). Further measurements of the viral RNA level (Fig. 2B) and viral titer in the supernatants (Fig. 2C) also showed similar results. In our previous study, GBP5 was shown to be essential for anti-RSV function from interferon-gamma (IFN-γ) (15). On this basis, the function of KRT9 on anti-RSV was also measured with or without IFN-γ treatments in RSV-targeting cells. In A549 infection, KRT9 knockdown did not affect RSV-N expression without IFN-γ treatment (Fig. 2D, lanes 2 and 5); however, it was affected with IFN-γ treatment (Fig. 2D, lanes 3 and 6). Similar results were obtained while measuring the viral RNA level (Fig. 2E) and viral titer (Fig. 2F). Additionally, primary bronchial epithelial cells were introduced in the abovementioned assay. Although with a low expression level of KRT9, the results were still consistent with those of the above experiments (Fig. 2G through I). On the other hand, HEK293T cells were transfected with combined GBP5-Flag and KRT9-Myc before RSV infection. After 48 hours post-infection, we found that KRT9-Myc did not decrease RSV-N expression individually (Fig. 2J, lanes 2 and 5). Furthermore, KRT9-Myc expression enhanced the decline in RSV-N expression (Fig. 2J, lanes 3 and 6). Similar results were also obtained while measuring the viral RNA level (Fig. 2K) and viral titer (Fig. 2L). These results indicate that KRT9 was unable to inhibit RSV replications individually, but played an important role in the GBP5 anti-RSV process.

### GBP5 is an adapter for interactions between SH and KRT9

**Fig 3:**
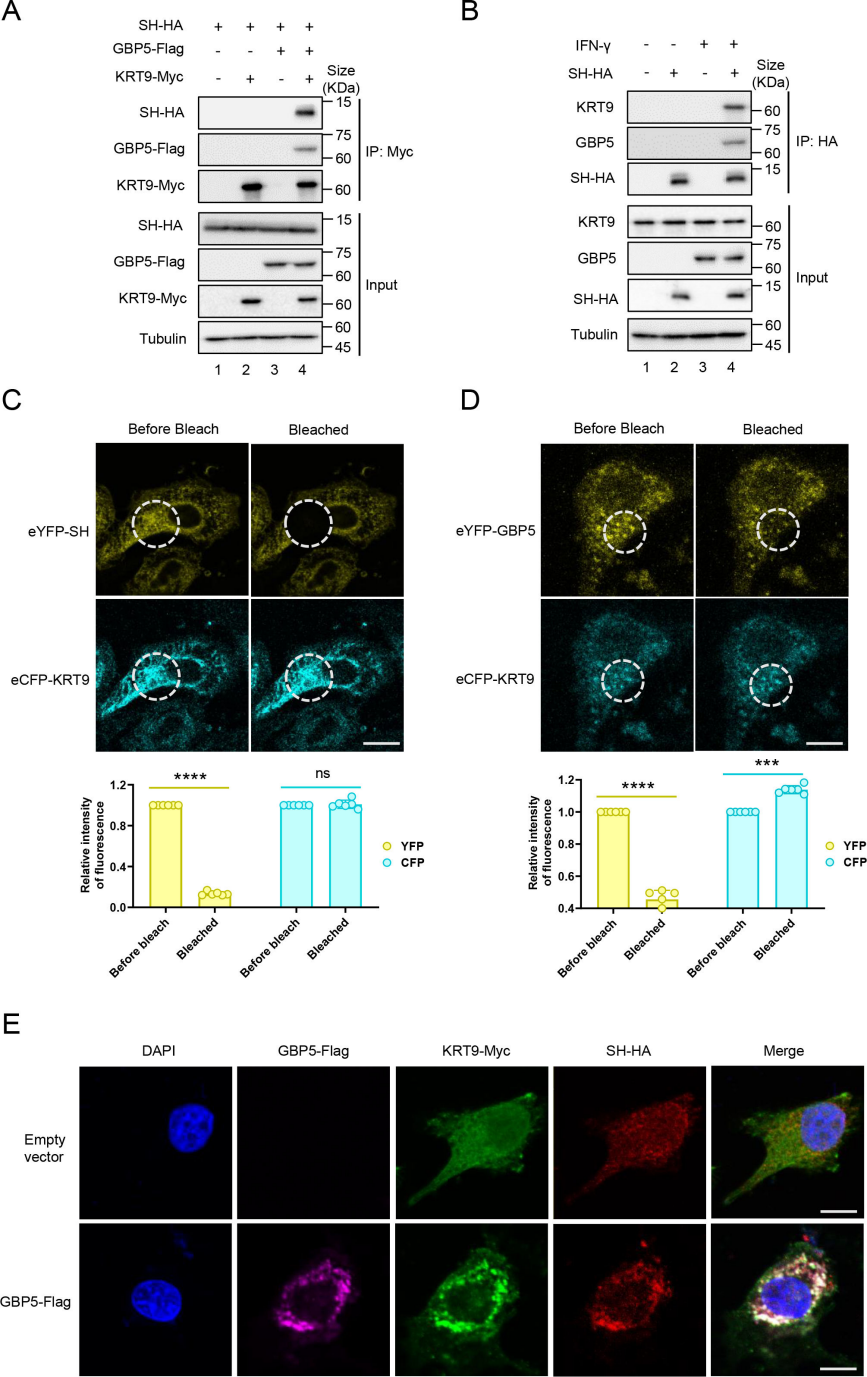
GBP5 but not SH interacts with KRT9 directly. (**A**) HEK293T cells transfected with SH-HA, KRT9-Myc, GBP5-Flag, or empty vector (VR1012) as annotations in the figure for 24 hours were harvested and lysed. And then, Co-IP assay was performed with protein-G agarose and anti-Myc antibody. The inputs and elution were analyzed by IB. (**B**) A549 cells transfected with SH-HA or empty vector by Lipofectamine 3000 for 24 hours were treated with or without IFN-γ (1,000 U/mL) as annotations in the figure for 24 hours. And then, cells were lysed, and then Co-IP assay was performed with protein-G agarose and anti-HA antibody. The elution was analyzed by IB. (**C and D**) Hela cells transfected with eCFP-KRT9 and eYFP-SH (**C**) or eYFP-GBP5 (**D**) for 24 hours were fixed in 4% paraformaldehyde. After washing three times with PBS, FRET assay was performed with the Olympus FV 3000 confocal imaging system. Intensities of fluorescence in the bleach area were analyzed by ImageJ and presented as histograms by GraphPad. The significance was determined by a two-tailed *t*-test: ***P < 0.001; ****P < 0.0001; ns means no significance. (**E**) Hela cells transfected with KRT9-Myc, SH-HA, and GBP5-Flag or empty vector for 24 hours were fixed in 4% paraformaldehyde. After 0.25% Triton-X100 treatment and 10% FBS blocking, rabbit anti-HA antibody and mouse anti-Myc antibody were used to incubate cells for 2 hours. And then, cells were washed three times with PBS and incubated with goat anti-Mouse IgG (H + L) Highly Cross Adsorbed Secondary Antibody, Alexa Fluor Plus 488, Goat anti-Rabbit IgG (H + L) Cross-Adsorbed Secondary Antibody, Alexa Fluor 568 and Rat anti-Flag monoclonal antibody, and Alexa Fluor Plus 647 for 1 hour. After being washed with cold PBS and treated with 5 µg/mL DAPI for 1 minute, cells were analyzed by the Olympus FV 3000 confocal imaging system. These data are representative of three independent experiments.

To investigate the mechanism of KRT9 function on the GBP5 anti-RSV process, Co-IP assays were performed between KRT9-Myc, GBP5-Flag, and RSV-SH-HA in HEK293T cells. The results showed us that KRT9-Myc did not bind to SH-HA without GBP5-Flag expression (Fig. 3A, lanes 1 and 2); however, expression and immunoprecipitation of GBP5-Flag pulldown the three proteins, KRT9-myc, SH-HA, and GBP5-Flag (Fig. 3A, lanes 3 and 4). A similar assay was performed in A549 cells. The results showed that IFN-γ treatment triggered endogenous GBP5 expression and interactions between SH-HA and endogenous KRT9 and with GBP5 (Fig. 3B). In our previous study, we confirmed the direct interaction between GBP5 and RSV-SH (15). To further reveal details of the interactions between KRT9, GBP5, and RSV-SH, fluorescence resonance energy transfer assays were performed with eCFP-KRT9, eYFP-SH, and eYFP-GBP5, respectively, in Hela cells. We found that when we bleached the fluorescence of eYFP-SH, eCFP-KRT9 fluorescence maintained its original intensity (Fig. 3C); however, when we bleached the fluorescence of eYFP-GBP5, the intensity of eCFP-KRT9 fluorescence increased (Fig. 3D). These results suggest that KRT9 binds to GPB5 directly but not to RSV-SH. In addition, GBP5-Flag, but not the empty vector (VR1012), triggered the perinuclear colocalization of KRT9-Myc and SH-HA in Hela cells (Fig. 3E). Thus, our results suggest that GBP5 mediated the interaction between KRT9 and RSV-SH.

### C-terminal of GBP5 and fragment 202–302 of KRT9 are essential for their interaction

**Fig 4:**
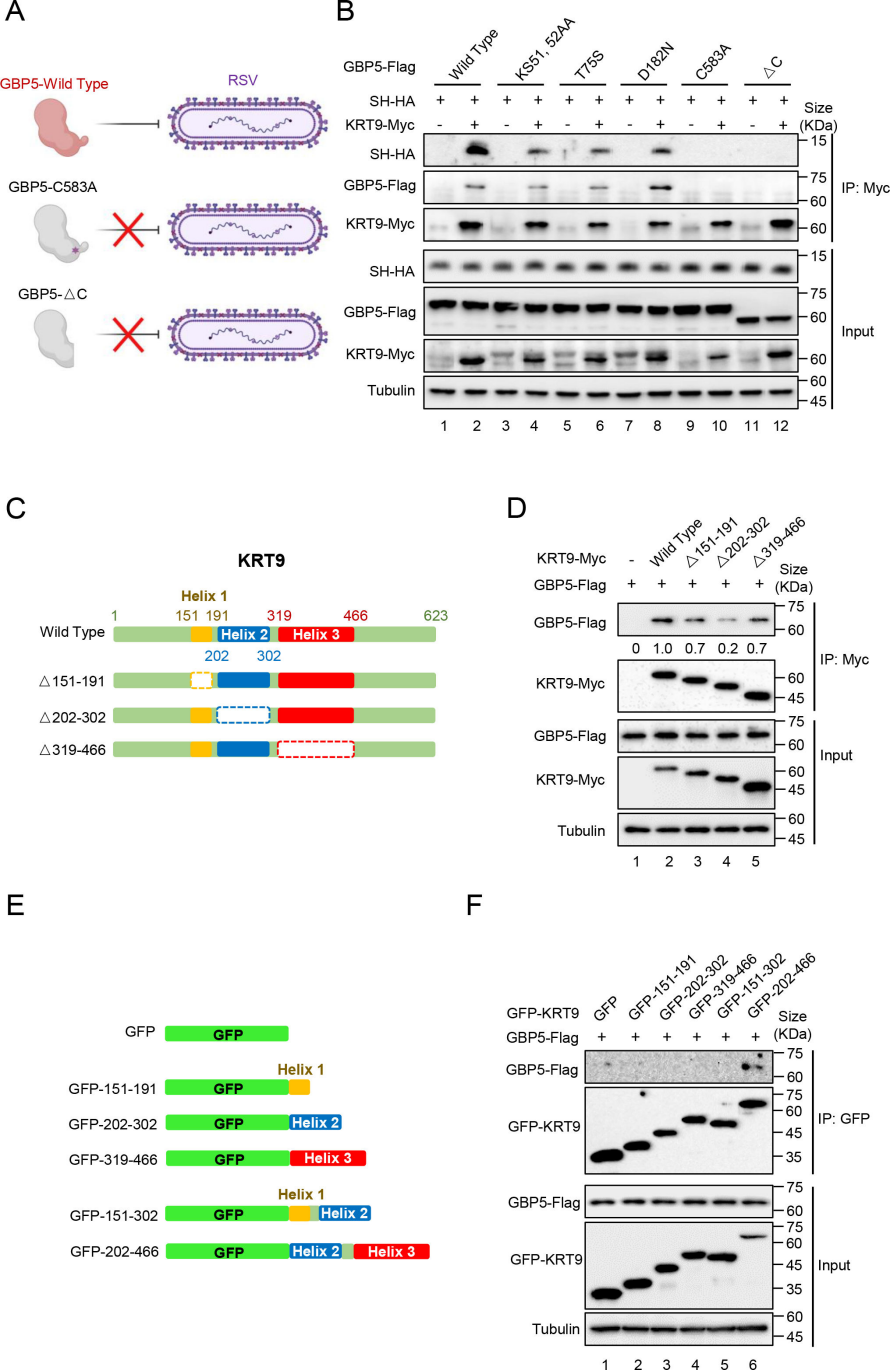
Identifications of the domain for the interactions between KRT9 and GBP5. (**A**) Schematic diagram of the loss of RSV inhibition by the C583A mutant or △C truncation of GBP5 (15). (**B**) HEK293T cells transfected with SH-HA, GBP5-Flag, and its mutants combined with KRT9-Myc or empty vector as annotations in the figure for 24 hours were harvested and lysed. And then, Co-IP was performed with protein-G agarose and anti-Myc antibody. The inputs and elution were analyzed by IB. (**C**) Schematic diagram of the truncations of KRT9. (**D**) HEK293T cells transfected with GBP5-Flag and wild-type, and truncations of KRT9-Myc or empty vector for 24 hours were harvested and lysed. And then, Co-IP was performed with protein-G agarose and anti-Myc antibody. The inputs and elution were analyzed by IB. The intensity of the GBP5-flag was analyzed by ImageJ and calibrated by tubulin. (**E**) Schematic diagram of a potential key motif of KRT9 conjugated with a GFP. (**F**) HEK293T cells transfected with GBP5-Flag and different fragments of KRT9 or the empty vector contained a GFP for 24 hours were harvested and lysed. And then, Co-IP was performed with protein-G agarose and anti-Myc antibody. The inputs and elution were analyzed by IB. These data are representative of three independent experiments.

In our previous study, C-terminal-deficient GBP5 and C583A mutant of GBP5 could not inhibit RSV replication (Fig. 4A) (15). These two mutants of GBP5 maintained the interactions with RSV-SH (data not shown). To investigate the reason for this and reveal the mechanism of interactions between KRT9 and GBP5, a Co-IP assay was performed with KRT9-Myc, SH-HA, and wild-type or mutants of GBP5-Flag. The results showed that different types of GBP5-Flag, which had the function on anti-RSV (15), except for C583A and △C, maintained the interaction with KRT9-Myc and mediated the interaction between KRT9-Myc and RSV-SH-HA (Fig. 4B). For the essential domain on KRT9, the secondary structure of KRT9 was predicted on the SWISS-MODEL website. According to this prediction, truncational mutants were constructed (Fig. 4C), and the Co-IP assay was performed. The results indicated that △202–302 of KRT9-Myc was weakly bound to GBP5-Flag compared with the other types (Fig. 4D). The fragments of KRT9 including these secondary structures were constructed following a GFP tag (Fig. 4E). To confirm the results of the previous study, Co-IP assay was performed with these fragments of KRT9 and GBP5-Flag. The 202–302 fragment of KRT9-Myc failed to bind to GBP5; however, the 202–466 fragment maintained the binding activity. These results suggested that the KRT9 202–302 fragment could be essential for GBP5 binding.

### GBP5 anti-RSV function is dependent on the interaction with KRT9

**Fig 5:**
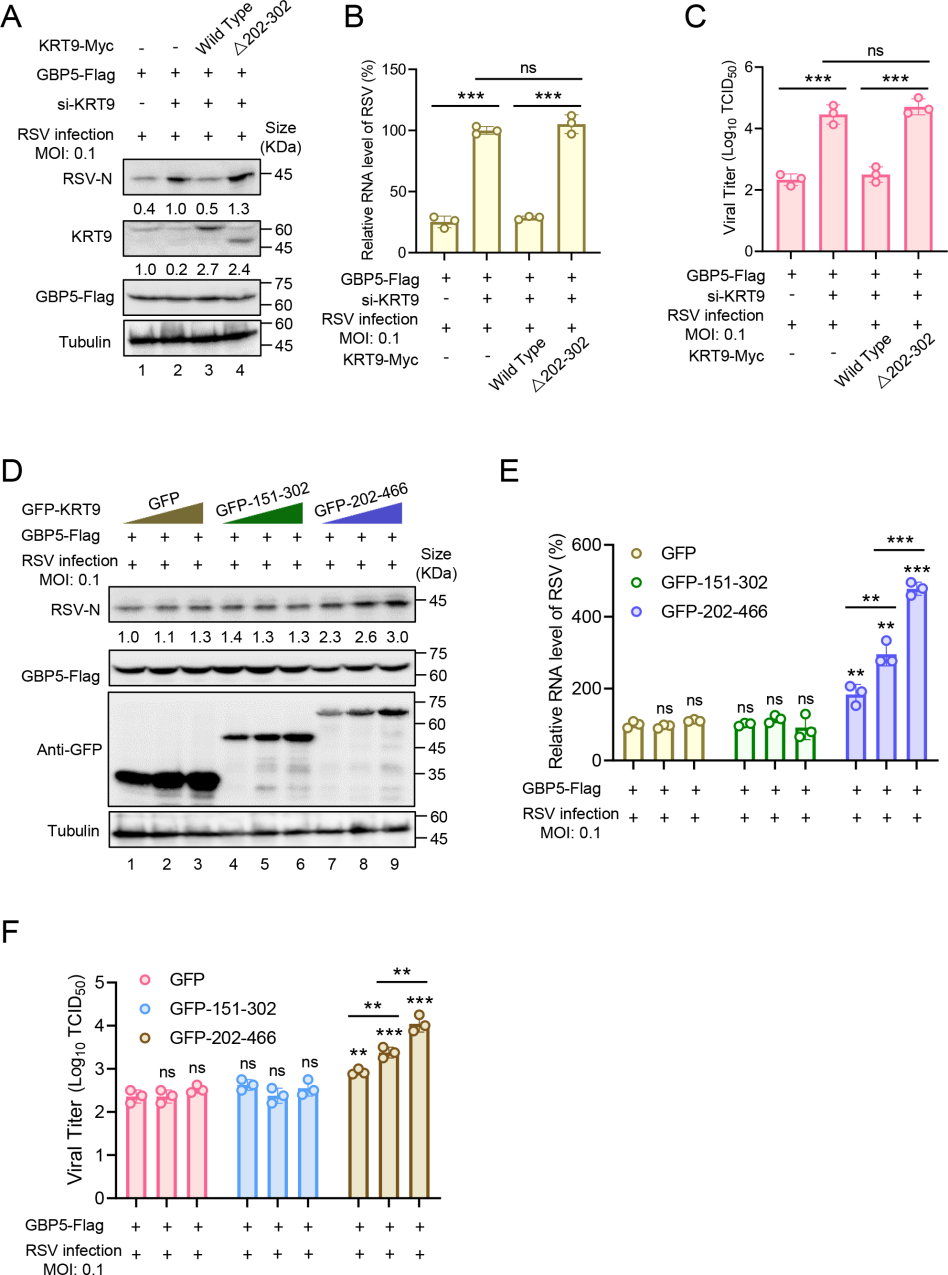
202–466 of KRT9 is required for GBP5 inhibiting RSV replication. (**A–C**) HEK293T cells transfected with siRNA by Lipofectamine RNAiMAX for 24 hours were transfected with GBP5-Flag, KRT9-Myc, or empty vector as annotation for 24 hours. And then, cells were infected with RSV at 0.1 MOI. After 48 hours post-infection, cells were harvested and detected by IB (**A**) and RT-qPCR (**B**). Viral titers in supernatants were measured by the endpoint dilution titer method (**C**). (**D–F**) HEK293T cells transfected with GBP5-Flag and serial doses of different domains of KRT9 conjugated with a GFP for 24 hours were infected with RSV at 0.1 MOI. After 48 hours post-infection, cells were harvested and detected by IB (**D**) and RT-qPCR (**E**). Viral titers in supernatants were measured by the endpoint dilution titer method (**F**). These data are representative of three independent experiments and shown as average ±SD (*n* = 3). Significance was determined by one-way ANOVA, followed by a Tukey multiple comparisons posttest. **P < 0.01; ***P < 0.001; ns means no significance. The intensities of KRT9 and RSV-N were analyzed by ImageJ and calibrated by tubulin.

For further revealing the role of KRT9 in the GBP5 anti-RSV process, endogenous KRT9 knockdown HEK293T cells were overexpressed with the wild-type or a 202–302 deletion mutant of KRT9 and infected with RSV at 0.1 multiplicity of infection. After 48 hours post-infection, we found that overexpression of wild-type KRT9 maintained the suppression of RSV replication (Fig. 5A, lanes 1–3), but the △202–302 mutant failed (Fig. 5A, lane 4). Similar results were found at the RSV RNA level in cell lysates (Fig. 5B) and viral titers in supernatants (Fig. 5C). Conversely, we hijacked GBP5 through the overexpressing fragment 202–466 of KRT9 and detected its anti-RSV function. We found that fragment 202–466, which interacted with GBP5-Flag (Fig. 4F), but not 151–302 of KRT9, weakened the anti-RSV function of GBP5 in a dose-dependent manner (Fig. 5D). Similar results were found at the RSV RNA level in cell lysates (Fig. 5E) and viral titers in supernatants (Fig. 5F). These results suggest that an interaction between KRT9 and GBP5 was required for their antiviral activity. A unitary motif of KRT9 is not enough to bind with GBP5 to affect its anti-RSV function.

### KRT9, without its type II keratin-binding partners, contributes to the GBP5 anti-RSV process

**Fig 6:**
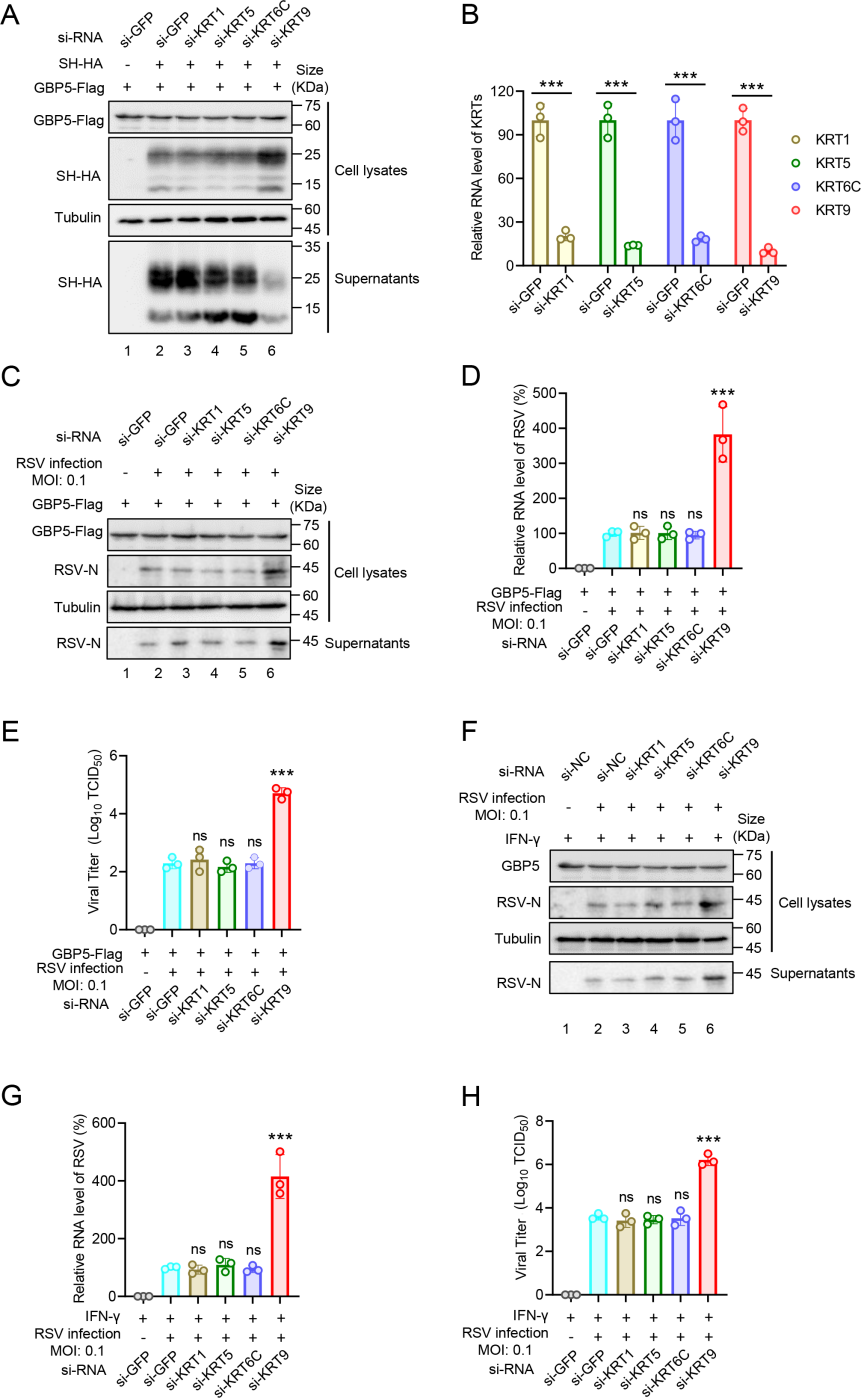
KRT9 but not other KRTs is involved in the GBP5 antiviral process. (**A and B**) HEK293T cells transfected with siRNA by Lipofectamine RNAiMAX for 24 hours were transfected with GBP5-Flag and SH-HA or empty vector as annotation. After 24 hours post-transfection, cells and supernatants were harvested and detected by IB (**A**) and RT-qPCR (**B**), respectively. (**C-E**) HEK293T cells transfected with siRNA by Lipofectamine RNAiMAX for 24 hours were transfected with GBP5-Flag for 24 hours. And then, cells were infected with RSV at 0.1 MOI as annotation. After 48 hours post-infection, cells and supernatants were harvested and detected by IB (**C**) and RT-qPCR (**D**), respectively. Viral titers in supernatants were measured by the endpoint dilution titer method (**E**). (**F–H**) A549 cells transfected with siRNA by Lipofectamine RNAiMAX for 24 hours were treated with IFN-γ (1000 U/mL) for 24 hours. And then, cells were infected with RSV at 0.1 MOI as annotation. After 48 hours post-infection, cells and supernatants were harvested and detected by IB (**F**) and RT-qPCR (**G**), respectively. Viral titers in supernatants were measured by the endpoint dilution titer method (**H**). These data are representative of three independent experiments and shown as average ±SD (*n* = 3). Significance was determined by one-way ANOVA, followed by a Tukey multiple comparisons posttest. ***P < 0.001; ns means no significance.

KRT9 has three potential type II keratin-binding partners, KRT1, KRT5, and KRT6C (27–29). For further investigation, we silenced the expressions of KRT1, KRT5, KRT6C, and KRT9 and confirmed GBP5 function on the RSV-SH protein. The results showed that KTR9, but not its partners, affected GBP5 function (Fig. 6A). Simultaneously, the effects of siRNAs used for silencing KRTs were confirmed (Fig. 6B). GBP5 function on anti-RSV was detected after silencing different KRTs. We found that silencing KRT9, but not its partners, increased the viral protein abundance (Fig. 6C) and viral-RNA (Fig. 6D) in cells and the viral titer in supernatants (Fig. 6E). Furthermore, silencing KRT9 had a similar effect on IFN-γ and inhibited RSV replication (Fig. 6F through H). These results suggest that the role of KRT9 in the anti-RSV process was independent of its function on epidermolytic palmoplantar keratoderma.

## DISCUSSION

**Fig 7:**
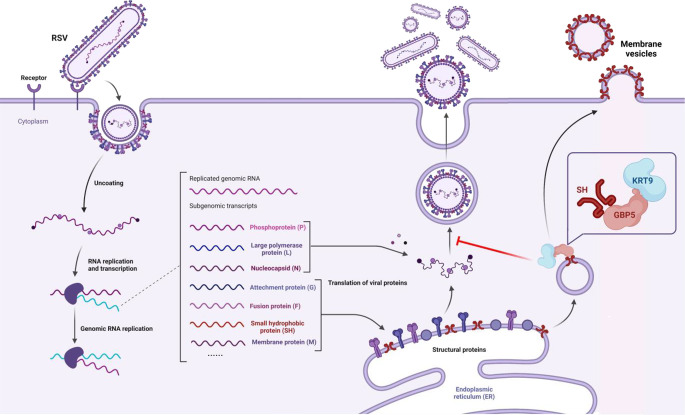
Schematic diagram of the participation of KRT9 In the process of inhibition of RSV replication by GBP5. In the RSV replication process, SH protein, as a structural protein, participated in the viral assembly. However, GBP5 hijacks SH protein, sends it into membrane vesicles through binding KRT9, triggers the excessive secretion of SH protein, and finally inhibits the viral assembly. The schematic diagram was drawn using Biorender, and the agreement number is NE27OEJKHH.

Severe acute lower respiratory tract infections caused by RSV have caused a substantial burden of disease worldwide in recent years (5). Despite the availability of vaccines, the possibility of immune escape via mutations in RSV remains a concern. With the lack of specific drugs targeting RSV, research should focus on the natural immune interaction mechanism between RSV and the host. In our previous study, we showed that GBP5 inhibited RSV replication via triggering the excessive secretion of RSV-SH protein by microvesicles. However, GBP5 transport by vesicles or fibers was not reported. For revealing this mechanism of RSV-SH transport mediated by GBP5, we performed a series of affinity-mass spectroscopy analyses and found that KRT9 played an indispensable role in this process via RNA interference (Fig. 1). Keratins are major cytoskeletal components in epithelial cells, are reported to be transported on the actin cytoskeleton (38–42), and are composed of a transport network (43, 44). Further investigation suggested that keratins were transported by kinesin-1 (45). KRT9, which is responsible for epidermolytic palmoplantar keratoderma, has been mainly reported to be primarily expressed in the suprabasal cells and eccrine sweat-gland ducts in the palms and soles (46, 47). However, recently, KRT9 has been shown to occur in many other tissues and has also been associated with bladder cancer (34), gastric cancer (35), TAMG (+) thymomas (37), primary melanoma, and benign nevi (36). On the other hand, in the GeneCards database, the KRT9 protein is shown to be expressed in respiratory organs, including the nasopharynx, nasal respiratory epithelium, and lungs. Overall, we did detect a low expression level of KRT9 in primary bronchial epithelial cells (Fig. 2G). Therefore, KRT9, as type I keratin, was suggested to facilitate GBP5 in promoting RSV-SH protein transport in this study. GBP5 and IFN-γ were found to have lost their functions with decreased KRT9 expression (Fig. 1 and 2). Furthermore, the Co-IP and FRET assays showed that KRT9 bonded with GBP5 but not with RSV-SH directly (Fig. 3). Considering the direct interaction between GBP5 and RSV-SH (15), these results suggest that GBP5 could be a mediator that binds to RSV-SH and KRT9, and KRT9 promotes RSV-SH transport (Fig. 7). Conversely, KRT9 identification explained the loss of function of GBP5-C583A and GBP5-△C on RSV-SH (Fig. 4A and B).

While identifying the GBP5 binding domain on KRT9, the △202–302 but not △319–466 mutant was significantly weakly bound to GBP5 than the wild-type KRT9 (Fig. 4C, D and 5A through C). However, GFP-202–302 could not interact with GBP5 individually. With the presence of helix 3 (319–466), GFP-202–466 bonded to GBP5 successfully (Fig. 4E, F and 5D through F). These results suggest that helix 2 (202–302) may be essential for the interaction with GBP5; however, it needs other KRT9 domains to stabilize its suitable conformation. Conversely, we also detected the function of the potential type II partners of KRT9 in the GBP5 anti-RSV process. However, we did not find any positive effect on viral replication. (Fig. 6). Our findings do not suggest that KRT9 can perform its function without any other type II partner, and various tissues in which it occurs require further in-depth and focused research. Overall, this study is the first to characterize the synergistic effects of KRT9 and GPB5, which act as restriction factors against RSV.

## MATERIALS AND METHODS

### Plasmid construction

The cDNA of KRT9 was reverse-transcribed from the mRNAs, which were extracted from HEK293T cells, and then constructed into VR1012 between *SalI* and *BamHI* sites with a myc tag at its C terminal. GBP5 with an HA tag or flag tag, mutants and truncations of GBP5, GBP5-YFP, and SH-HA expression vectors were described in a previous study (15). For CFP-KRT9 and YFP-SH expression vectors, the fragments of KRT9 and SH were inserted into pECFP-C1 or pEYFP-C1 between *SalI* and *BamHI* sites, respectively. Deletion mutants of KRT9 expression vectors were constructed by mutagenesis with primers listed in Table 1. For truncations of KRT9 conjugated with GFP expression vectors, the partial fragments of KRT9 were inserted into pEGFP-C1 between *SalI* and *BamHI* sites, respectively.

**TABLE 1:** Primers used in this study

Primer name	Sequence (5’−3’)	Enzyme site	Purpose
XhoI-KRT9-CFP-F	CCCTCGAGATATGAGCTGCAGACAGTTCTC	XhoI	KRT9-CFP
KRT9-CFP-EcoRI-R	CGGAATTCCTAGGAATGGGATGATTTTC	EcoRI	KRT9-CFP
SalI-SH-YFP-F	GCGTCGACGAAAATACATCCATAACAATAG	SalI	SH-YFP
SH-YFP-BamHI-R	CGGGATCCCTATGTGTTGACTCGAGC	BamHI	SH-YFP
KRT9-△151–191-F	GGTGGTATTCTGACTAAGGGACCTGCTG	None	KRT9-△151–191
KRT9-△151–191-R	TAGTCAGAATACCACCATCACCTCC	None	KRT9-△151–191
KRT9-△202–302-F	TATCCAGAAGAACTACAACAGTGGAGATGTC	None	KRT9-△202–302
KRT9-△202–302-R	TGTAGTTCTTCTGGATAGCAGCAGGTC	None	KRT9-△202–302
KRT9-△319–466-F	CTCCTGGCAAAGATTCCTCCGGAGCTG	None	KRT9-△319–466
KRT9-△319–466-R	AATCTTTGCCAGGAGCAACGTTTATC	None	KRT9-△319–466
SalI-Myc-KRT9-151-191-F	GCGTCGACATGGAACAAAAACTCATATCAGAAGAAGATCTAGCTAATGAGAAGAGCACCATG	SalI	KRT9-151-191
KRT9-151-191-BamHI-R	CGGGATCCTCACTTGTCGTACCAATCCTGG	BamHI	KRT9-151-191
SalI-Myc-KRT9-202-302-F	GCGTCGACATGGAACAAAAACTCATATCAGAAGAAGATCTATCCCCTTATTATAACACTATTG	SalI	KRT9-202-302
KRT9-202-302-BamHI-R	CGGGATCCTCACTGCCCAGTCAGCTGAC	BamHI	KRT9-202-302
SalI-Myc-KRT9-319-466-F	GCGTCGACATGGAACAAAAACTCATATCAGAAGAAGATCTACTCACCAAGACCCTCAATG	SalI	KRT9-319-466
KRT9-319-466-BamHI-R	CGGGATCCTCATTCAAAGTCTTCCTGGCCTC	BamHI	KRT9-319-466
LTF-RT-F	ACCGAGAACACCGAGGAG	None	qPCR-LTF
LTF-RT-R	CCATCATCCGCCTTCTGG	None	qPCR-LTF
ACTB-RT-F	CCACACCTTCTACAATGAGC	None	qPCR-ACTB
ACTB -RT-R	GTACATGGCTGGGGTGTTG	None	qPCR-ACTB
TUFM-RT-F	GTGGGGCTAAGTTCAAGAAG	None	qPCR-TUFM
TUFM-RT-R	CATGACCCGGGCAGTCTG	None	qPCR-TUFM
AHSG-RT-F	GAGCATGCTGTCGAAGGAG	None	qPCR-AHSG
AHSG-RT-R	GGGCAGTCTTGGCACACC	None	qPCR-AHSG
HBA1-RT-F	GTGCGGAGGCCCTGGAG	None	qPCR-HBA1
HBA1-RT-R	GCGCGTCGGCCACCTTC	None	qPCR-HBA1
ITIH2-RT-F	TTCTCTGTGATTGATTTCAACC	None	qPCR-ITIH2
ITIH2-RT-R	AGTGCTTCGTTGATGTTTGTG	None	qPCR-ITIH2
ITIH3-RT-F	TGAAGAGCATGGAGGATAAAG	None	qPCR-ITIH3
ITIH3-RT-R	TGATGACAATGGAGGTGCTC	None	qPCR-ITIH3
ITIH4-RT-F	GGTCAAGCACCTGCAGATG	None	qPCR-ITIH4
ITIH4-RT-R	AACCGGATGTGAGCCTTGG	None	qPCR-ITIH4
GSN-RT-F	AGCTTCAACAATGGCGACTG	None	qPCR-GSN
GSN-RT-R	CACTCCGCTCGTTGTCCC	None	qPCR-GSN
LDHA-RT-F	GGCAGCCTTTTCCTTAGAAC	None	qPCR-LDHA
LDHA-RT-R	GCTGGACCAAATTAAGACGG	None	qPCR-LDHA
C3-RT-F	GATGACAGAGGATGCCGTC	None	qPCR-C3
C3-RT-R	TCTCCCACTGCTCCGTTTC	None	qPCR-C3
C4A-RT-F	AAGAGCTGTGGCCTCCATC	None	qPCR-C4A
C4A-RT-R	GGAGAAGAGCAGGTTGATAC	None	qPCR-C4A
SLC1A5-RT-F	GAAGAGAGGAATATCACCGG	None	qPCR-SLC1A5
SLC1A5-RT-R	TAAGCAGCTCCCCTTCAGG	None	qPCR-SLC1A5
CFL1-RT-F	GAACATCATCCTGGAGGAG	None	qPCR-CFL1
CFL1-RT-R	TTGGTCTCATAGGTTGCATC	None	qPCR-CFL1
DMBT1-RT-F	ATAGTGAAGACGCTGGTGTC	None	qPCR-DMBT1
DMBT1-RT-R	GTCACCTCCATTCACCAGC	None	qPCR-DMBT1
SERPINF1-RT-F	TCCTGACGGGCAACCCTC	None	qPCR-SERPINF1
SERPINF1-RT-R	GAAGTGCGCCACACCGAG	None	qPCR-SERPINF1
THBS1-RT-F	CAGCTGTACATCGACTGTG	None	qPCR-THBS1
THBS1-RT-R	TGGAAATTGTCATTGACGCC	None	qPCR-THBS1
HSPA8-RT-F	GGAAATTGCAGAAGCCTACC	None	qPCR-HSPA8
HSPA8-RT-R	GAGACCAGCAATAGTTCCAG	None	qPCR-HSPA8
RAN-RT-F	AGGAGAAATTCGGTGGACTG	None	qPCR-RAN
RAN-RT-R	TCACACACTCGTACCAGATC	None	qPCR-RAN
KRT2-RT-F	CAGGGGTATATCGACAGCC	None	qPCR-KRT2
KRT2-RT-R	GTGCGCTTATTGATTTCATCC	None	qPCR-KRT2
KRT9-RT-F	TGACCATGGAGAAGTCTGAC	None	qPCR-KRT9
KRT9-RT-R	GCAACGTTTATCTCCACATTG	None	qPCR-KRT9
FN1-RT-F	GGTGACACTTATGAGCGTC	None	qPCR-FN1
FN1-RT-R	CCAGGTGTCACCAATCTTG	None	qPCR-FN1
ALDOA-RT-F	ACCGAGAACACCGAGGAG	None	qPCR-ALDOA
ALDOA-RT-R	CCATCATCCGCCTTCTGG	None	qPCR-ALDOA
KRT1-RT-F	ATGTGGAGATTGACCCTGAG	None	qPCR-KRT1
KRT1-RT-R	CATTTTGTTTGCAGTACCTGG	None	qPCR-KRT1
KRT5-RT-F	CTGGAGCAGCAGAACAAGG	None	qPCR-KRT5
KRT5-RT-R	CTGCCTCCTGAGGTTGTTG	None	qPCR-KRT5
KRT6C-RT-F	AACATGCAGGACCTGGTGG	None	qPCR-KRT6C
KRT6C-RT-R	GTGAGAGTGTCTGCCTTGG	None	qPCR-KRT6C
GBP5-RT-F	CGCACTGGCAAATCCTACC	None	qPCR-GBP5
GBP5-RT-R	GAACTAATGTGTGATTTGGCC	None	qPCR-GBP5
RSV-N-RT-F	TGCAGGGCAAGTGATGTTAC	None	qPCR-RSV-N
RSV-N-RT-R	TTCCATTTCTGCTTGCACAC	None	qPCR-RSV-N

### Cell culture and viruses

HEK293T (American Type Culture Collection [ATCC], Manassas, VA, USA, catalog no. CRL-11268), Hela cells (ATCC, catalog no.CRM-CCL-2), Hep-2 (ATCC, catalog no. CCL-23), and A549 (ATCC, catalog no. CCL-185) cells were cultured as monolayers in Dulbecco’s modified Eagle’s medium (DMEM) (Hyclone, Logan, UT, USA) supplemented with 10% heat-inactivated (56°C, 30 minutes) fetal calf serum (FCS, GIBCO BRL, Grand Island, NY, USA) and maintained at 37°C with 5% CO_2_ in a humidified atmosphere. RSA A2 strain was a gift from Jilin University College of Life Science professor Chunlai Jiang.

### Virus titration

For titration, as descripted in previous study (48), Hep-2 cells were infected with 1:10 dilutions of the supernatants including viruses in the presence of 2% FBS. After 4–5 days of incubation in 7% CO_2_ at 37°C, end point dilution titer (TCID_50_) was determined by crystal violet staining of the monolayers and calculated by the Reed–Muench formula.

### Transfection and infection

Plasmid transfections were performed with Lipofectamine 3000 (Invitrogen, Carlsbad, CA, USA, catalog no. L3000-008) according to the manufacturer’s instruction. For siRNA(Table 2) transfections, Lipofectamine RNAiMAX (Invitrogen, catalog no. 13778150) was used according to its official instructions. For RSV infection, A549 or HEK29T cells were seeded in 6-well plates and grown to 70% confluence. After washing with PBS, cells were incubated with RSV in 37°C for 2 hours, and then supernatants were replaced by fresh medium containing 10% FBS and maintained in 37°C and 5% CO_2_.

**TABLE 2:** siRNA used in this study

siRNA name	Sequence (5’−3’)
si-GFP	AUCACCCUGCAAACUUGUUTT
si-LTF	ACAAAUGCAACGUCUCCAGTT
si-ATCB	ACAUAGGAAUCCUUCUGACTT
si-TUFM	UAUUCUUAACAUAAUCUGCTT
si-AHSG	UAACAGGCACAUAUAAGCUTT
si-HBA1	AAGCAUGGCCACCGAGGCUTT
si-ITIH2	UCGAACUGCACCUUUGCUCTT
si-ITIH3	UUGAGGUGCAAAGAAGUGCTT
si-ITIH4	UGGAAAGUGAUGUUCUGUGTT
si-GSN	GAUUUUCCCAUCUUUGCCGTT
si-LDHA	AUUACUGCUUUAAUCACAGTT
si-C3	AGUAUUGGUGAACUUUGAATT
si-C4A	UCUAACUGCAUUUCAUCAATT
si-SLC1A5	UCUACAUUGAGGACGGUACTT
si-CFL1	ACUAUUGCGGUUAGAAGUUTT
si-DMBT1	UCAAUGAUCCAUCAAAGAUTT
si-SERPINF1	AUUGUAUGCAUUGAAACCUTT
si-THBS1	CAUCAAUAUCCUGAUUGUUTT
si-HSPA8	AUCAACCUCUUCAAUGGUGTT
si-RAN	ACAUUGAACUUAAUAGGUCTT
si-KRT2	UCAACUUUCACGUUGAGAGTT
si-KRT9	UGAUUCUCGAUGUCCUUUCTT
si-FN1	AGAUAGUGAUGUUAUACUGTT
si-ALDOA	GCAAGUUCCUGGCACAGGATT
si-KRT1	CAACCGAGAUUGAUCACUCTT
si-KRT5	AUCCAUCAGUGCAUCAACCTT
si-KRT6C	AUACAAGGCUCUCAGGAAGTT

### RNA extraction and RT-qPCR

For RT-qPCR, viral or cell RNA was extracted from supernatants or cells with TRIzol reagent (Invitrogen, catalog no. 15596018), diethyl pyrocarbonate (DEPC)-treated water, and RNase inhibitor (New England BioLabs, Ipswich, MA, USA). The cDNA was generated by the Transcriptor First Strand cDNA Synthesis Kit (Roche, Basel, Basel-City, CH, catalog no. 04896866001) and oligo (dT) 18 primers or random primer according to the supplier’s instructions. RT-qPCR was carried out on an Mx3005P instrument (Agilent Technologies, Stratagene, La Jolla, CA, USA) with the FastStart Universal SYBR Green Master (Roche, catalog no. 04913914001) and designed primers targeting the conserved region sequences of RSV-N, GBP5, KRT1, KRT5, KRT6C, and KRT9 containing in this study. The RT-qPCR assay was performed in a 20 µL volume as per the manufacturer’s instructions. Amplification of the target fragment was carried out as follows: 95°C for 2 minutes, followed by 40 cycles of 95°C for 15 seconds, 55°C for 15 seconds, and 68°C for 20 seconds. All primers for qPCR are presented in Table 1.

### Immunoblotting analysis

Cells were harvested at 12,000 rpm for 2 minutes. Lysed in lysis buffer (50 mM Tris–HCl [pH 7.8], 150 mM NaCl, 1.0% NP-40, 5% glycerol and 4 mM EDTA), and 4 × loading buffer (0.08M Tris [pH 6.8], 2.0%SDS, 10% glycerol, 0.1 M dithiothreitol, and 0.2% bromophenol blue). The lysates were heated at 100 ° C for 30 minutes and centrifuged at 12,000 rpm for 10 minutes. Total cell lysates were separated to SDS-PAGE and transferred onto polyvinylidene fluoride (PVDF) membranes. After blocking with 5% skim milk powder in Tris-buffered saline–Tween (TBST) for 1 hour at room temperature. The membranes were incubated with the indicated primary antibodies overnight at 4°C and then incubated with HRP-conjugated secondary antibodies (Jackson Immunoresearch, West Grove, NJ, USA, catalog no. 115–035-062 for anti-mouse and 111–035-045 for anti-rabbit). We used an ultrasensitive ECL Chemiluminescence Detection Kit (Proteintech, Rosemont, IL, USA, catalog no. B500024) to make the bands on membranes visualized. Antibodies used in this study are as follows: anti-Flag mAb (Sigma, catalog no. F1804), anti-tubulin mAb (Abcam, Cambridge, Cambridgeshire, UK, catalog no. ab11323), anti-hemagglutinin (anti-HA) pAb (Invitrogen, catalog no. 71–5500), anti-Myc pAb (Proteintech, Rosemont, IL, USA, catalog no. 16286–1-AP), anti-GFP mAb (Abcam, catalog no. ab1218), anti-RSV-N mAb (GeneTex, Irvine, CA, USA, catalog no. GTX636648), anti-GBP5 pAb (Sangon Biotec, Shanghai, CHN, catalog no. D222408-0100), and anti-KRT9 mAb (Sangon Biotec, catalog no. D194651).

### Co-Immunoprecipitation (co-IP) assay

The co-IP assays were performed as previously reported (15). HEK293T cells transfected with expression vectors for 48 hours as described were harvested and washed twice with cold PBS, followed by disruption with lysis buffer (PBS containing 1% Triton X-100 and complete protease inhibitor cocktail [Roche]) at 4°C for 1 hour. Cell lysates were clarified by centrifugation at 10,000 x g for 30 minutes at 4°C. Appropriate antibodies and protein G-agarose beads (Roche, catalog no. 11243233001) were mixed with the pre-cleared cell lysates and incubated at 4°C for 4 hours on an end-over-end rocker. The reaction mixtures were then washed six times with cold wash buffer (20 mM Tris-HCl, pH 7.5, 100 mM NaCl, 0.1 mM EDTA, 0.05% Tween-20) and subsequently analyzed by immunoblotting.

### Confocal microscopy and fluorescence resonance energy transfer (FRET) analysis

For localization of KRT9, GBP5, and SH, Hela cells seeded in 6-well glass-bottom plates were transfected with KRT9-Myc, SH-HA and GBP5-Flag, or empty vector, respectively. Twenty-four hours after the post-transfection, cells were fixed in 4% paraformaldehyde at room temperature for 5 minutes, washed with PBS, permeabilized in 0.25% Triton X-100 for 5 minutes, washed in PBS, blocked in 10% FBS for 1 hour, and then incubated at room temperature for 2 hours with mouse anti-Myc antibody at 1:1000 and rabbit anti-HA pAb at 1:250. Following a wash, cells were incubated with goat anti-Mouse IgG (H + L) Highly Cross Adsorbed Secondary Antibody, Alexa Fluor Plus 488 (Invitrogen, catalog no. A11001), Goat anti-Rabbit IgG (H + L) Cross-Adsorbed Secondary Antibody, Alexa Fluor 568 (Invitrogen, catalog no. A-11011) and Rat anti-Flag monoclonal antibody, and Alexa Fluor Plus 647 (Invitrogen, catalog no. MA1-142-A647) at room temperature for 1 hour. After being washed with cold PBS and treated with 5 µg/mL DAPI (Sigma, catalog no. D8417) for 1 minute, cells were analyzed by using a laser scanning confocal microscope (Olympus FV 3000). For FRET, Hela cells seeded in 6-well glass-bottom plates were transfected with ECFP-KRT9 (1 ug) and EYFP-GBP5 or EYFP-SH (1 ug). After 24 hours for post-transfection, cells were fixed in 4% paraformaldehyde at room temperature for 5 minutes and washed with PBS three times. Fluorescent images of samples were then acquired with the Olympus FV 3000 confocal imaging system.

### Mass spectrometry (MS)

HEK293T cells transfected with empty vector (VR1012, 2 µg), GBP5-HA (2 µg), or SH-HA (2 µg) combined with GBP5-Flag (2 µg) for 48 hours were harvested from a 6-well plate and lysed in 0.4 mL lysis buffer. And then, a co-immunoprecipitation assay was performed with protein G agarose beads (30 µL/sample, Roche, catalog no. 11243233001) with the anti-HA antibody (4 uL/sample, Invitrogen, catalog no. 71–5500), and the elution was analyzed by mass spectrum. Mass spectrum analysis was performed by the national center for protein science (Beijing, CHN). In results analysis, the proteins, which appeared more both in GBP5-HA and SH-HA combined with GBP5-Flag than VR1012, were identified as the potential target genes and assessed in further investigations.

### Statistical analysis

The detailed statistical analysis has been described in figure legends. All data are expressed as the mean ± standard deviations (SDs). Statistical comparisons were made using Student’s *t*-test, one-way ANOVA, or repeated-measure ANOVA. Significant differences are indicated in figures as follows: *P < 0.05, **P < 0.01, and ***P < 0.001; ns stands for no significance.

## Data Availability

The mass spectrometry proteomics data have been deposited to the ProteomeXchange Consortium (http://proteomecentral.proteomexchange.org) via the iProX partner repository (49, 50) with the data set identifier PXD054621.

## SUPPLEMENTAL MATERIAL

Fig. S1 and S2 jvi.02029-24-s0001.pdf siRNA library-related data in this study.

ASM does not own the copyrights to Supplemental Material that may be linked to, or accessed through, an article. The authors have granted ASM a non-exclusive, world-wide license to publish the Supplemental Material files. Please contact the corresponding author directly for reuse.
