# Supplementary material for: KRT9 is required for GBP5 suppression of human respiratory syncytial virus
Source: J Virol. 2025 Jan 21;99(2):e02029-24. doi: 10.1128/jvi.02029-24 (PMC11852966; doi:10.1128/jvi.02029-24)
Supplement: Fig. S1 and S2 — siRNA library-related data in this study. [file jvi.02029-24-s0001.pdf]

Supplemental Material For

KRT9 is required for GBP5 suppression of human respiratory syncytial virus

Xinglong Qu[1, 3], Ziqi Zhu[3], Xinpei Zhou[3], Xuehan Wu[3], Xin Liu[3], Xiaoyu Sun[4], Jiayue Zhang[4], Guanyi Du[4], Runyu Xue[4], Qianhua Zhang[4], Wenyan Zhang [2, 3]* and Zhaolong Li [2, 3]*

[1]Respiratory department of the First Hospital of Jilin University, Changchun, Jilin, China; [2]Department of Infectious Diseases, Infectious Diseases and Pathogen Biology Center, Key Laboratory of Organ Regeneration and Transplantation of The Ministry of Education, The First Hospital of Jilin University, Changchun, Jilin, China; [3]Institute of Virology and AIDS Research, the First Hospital of Jilin University, Changchun, Jilin, China; [4]Clinical Medical School, Norman Bethune Health Science Center of Jilin University, Changchun, Jilin, China.

***Corresponding author:**
Institute of Virology and AIDS Research, the First Hospital of Jilin University. No 519 East Minzhu Avenue, Changchun 130021, P. R. China. E-mail: zhangwenyan@jlu.edu.cn and lizhaolong@jlu.edu.cn

This PDF file includes:
Supplemental Fig. S1-2

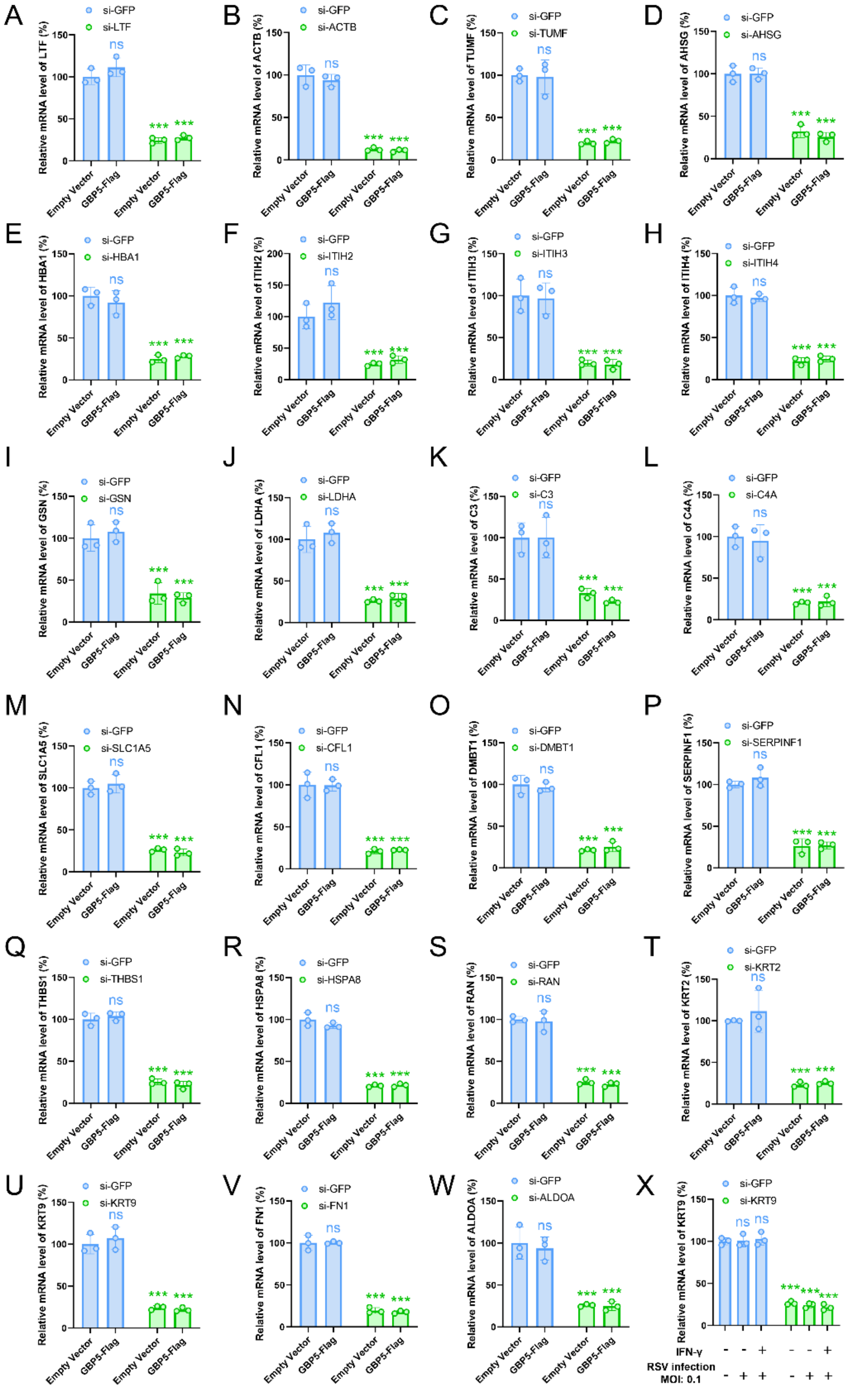

Fig. S1 siRNAs silenced target genes efficiently. RNAs were extracted from the cells in Fig. 1C (A-W) and Fig.2G (X), and then the target genes were analyzed by RT-qPCR. Data are representative of three independent experiments and shown as average ±SD (n = 3). Significance was determined by one-way ANOVA, followed by a Tukey multiple comparisons posttest. ***P < 0.001; ns means no significance.

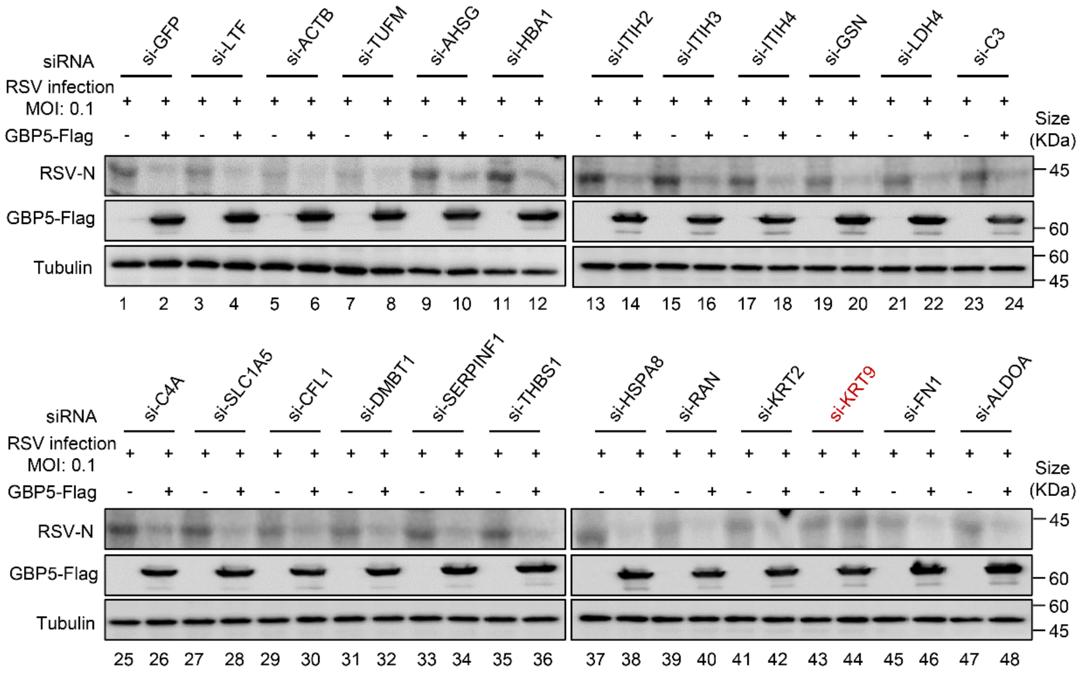

Fig. S2 KRT9 participated in GBP5 anti-RSV procession. HEK293T transfected with siRNA by Lipofectamine RNAiMAX for 24 hours were infected with RSV at 0.1 multiplicity of infection (MOI) for 48 hours. And then cells were harvested and analyzed by Immunoblotting (IB). Tubulin was detected as internal control.
